# Cryoballoon Left Atrial Roof Block Creation Leveraging the Left Atrial Roof Vein

**DOI:** 10.1002/ccr3.72321

**Published:** 2026-03-17

**Authors:** Yuhei Kasai, Takayuki Kitai, Junji Morita, Kei Murakami, Yumetsugu Munakata, Ryo Horita, Takuya Haraguchi, Jungo Kasai, Daisuke Hachinohe

**Affiliations:** ^1^ Department of Cardiology Sapporo Heart Center, Sapporo Cardiovascular Clinic Sapporo Japan; ^2^ Department of Clinical Engineering Sapporo Cardiovascular Clinic Sapporo Japan; ^3^ Toyota Technological Institute at Chicago Chicago Illinois USA

**Keywords:** atrial fibrillation, cryoballoon ablation, left atrial roof block, left atrial roof vein

## Abstract

The left atrial roof vein provides a stable and reproducible anchoring site for cryoballoon positioning, thereby facilitating effective and durable left atrial roof block creation in situations where conventional anchoring within the superior pulmonary veins is insufficient or unstable.

## 1 Clinical Question

How can stable balloon anchoring be achieved for left atrial roof block (LARB) creation during cryoballoon ablation when conventional pulmonary vein anchoring is inadequate?

## 2 Case Description

**FIGURE 1:**
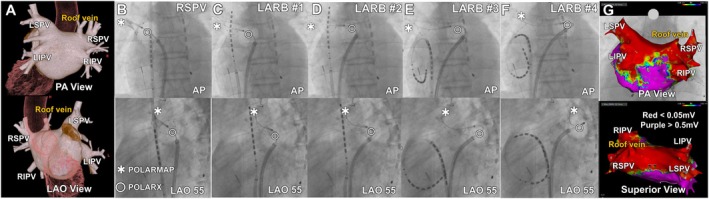
Preoperative computed tomography (CT) and intraoperative fluoroscopic images of the procedure. (A) Pre‐procedural CT demonstrated the roof vein, along with the left superior, left inferior, right superior, and right inferior pulmonary veins. (B) The POLARMAP catheter was positioned at the RSPV, and a 180‐s freeze achieved isolation. Phrenic pacing was performed via a duodecapolar catheter in the right subclavian vein to prevent phrenic injury. (C) The first cryoapplication targeted the left atrial roof, with the POLARMAP catheter advanced deeply into the upper RSPV and a 180‐s freeze delivered using the raise‐up technique. (D, E) The second (D) and third (E) cryoapplications targeted the left atrial roof, with the POLARMAP catheter anchored in the upper RSPV and sequential overlapping freezes delivered using the same rotational and sheath‐manipulation technique. (F) The fluoroscopic images show the fourth cryoapplication directed at the left atrial roof. During this freeze, the cryoballoon lost stable anchoring and collapsed toward the LSPV. Repositioning the POLARMAP catheter into the roof vein (RV) allowed successful completion of the freeze. (G) Post‐mapping using the CARTO3 system (Biosense Webster) confirmed durable pulmonary vein isolation and complete LARB creation. AP, anterior to posterior; PA, posterior to anterior; LAO, left anterior oblique; RSPV, right superior pulmonary vein; RIPV, right inferior pulmonary vein; LSPV, left superior pulmonary vein; LIPV, left inferior pulmonary vein; LARB, left atrial roof block.

**FIGURE 2:**
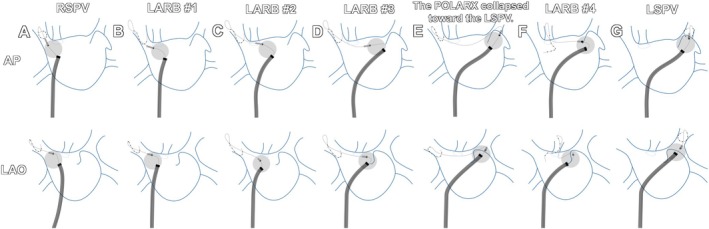
The schema drawing of cryoballoon‐guided LARB creation. (A) The POLARMAP catheter was positioned in the upper RSPV, and a 180‐s freeze achieved isolation (Figure 1B). (B) The first cryoapplication targeted the left atrial roof, with the POLARMAP catheter positioned in the upper RSPV (Figure 1C). (C, D) The second and third cryoapplications also targeted the left atrial roof, with the POLARMAP catheter similarly advanced deeply into the upper RSPV (Figure 1D,E, respectively). (E) The fourth cryoapplication was directed at the left atrial roof; however, shortly after the initiation of freezing, the cryoballoon lost its stable anchoring and collapsed toward the LSPV. (F) The POLARMAP catheter was repositioned into the RV, allowing successful completion of the fourth freeze. In the LAO view, the RV, located posterior to the RSPV (appearing more rightward), provided a more reliable anchoring point, enhancing stability on the LSPV side of the roof line (Figure 1F). (G) The lesion created by the fourth freeze was confirmed to be continuous with the freeze lesion for LSPV isolation. AP, anterior to posterior; LAO, left anterior oblique; RSPV, right superior pulmonary vein; LARB, left atrial roof block; LSPV, left superior pulmonary vein.

A 59‐year‐old man with symptomatic persistent atrial fibrillation (AF) underwent cryoballoon ablation under general anesthesia, a strategy associated with a lower risk of ablation‐induced stenosis than radiofrequency ablation (Figure 1A). After intracardiac echocardiography‐guided transseptal puncture, a steerable sheath (POLAR sheath; Boston Scientific, MA, USA) was advanced into the left atrium (LA). Following contrast‐confirmed occlusion, a 240‐s freeze was delivered to the left superior pulmonary vein (LSPV), and 180‐s freezes were applied to the remaining pulmonary veins using a POLARX cryoballoon (Boston Scientific) (Figures 1B and 2A). Subsequently, LARB creation was attempted. A mapping catheter (POLARMAP; Boston Scientific) was advanced deeply into the upper branch of the right superior pulmonary vein (RSPV) to anchor the cryoballoon, and sequential 180‐s freezes were applied using the raise‐up technique. The first three applications achieved temperatures below −50°C (Figures 1C–E and 2B–D). During the fourth application, however, balloon instability occurred with collapse toward the LSPV despite adjustment of the sheath deflection (Figure 2E). Repositioning the POLARMAP catheter into the upper branch of the LSPV resulted in insufficient cooling (> −40°C) and early termination. In contrast, anchoring the catheter within the roof vein (RV) achieved temperatures below −50°C, allowing successful completion of the final application (Figures 1F, 2F,G).

Post‐procedural mapping with a multielectrode catheter (Octaray; Biosense Webster, Irvine, CA, USA) confirmed durable pulmonary vein isolation (PVI), including the RV, and complete LARB creation (Figure 1G). No complications occurred, and the patient remained free from AF recurrence at 24‐month follow‐up.

## 3 Discussion

Adjunctive LARB creation significantly reduces arrhythmia recurrence compared with PVI alone [1]. Conventional techniques rely on RSPV or LSPV anchoring; however, this case instead leveraged the RV, an anatomical variant with an incidence of approximately 3% [2, 3]. Owing to its posterior orientation relative to the RSPV, the RV improved balloon stability on the left atrial roof, particularly on the LSPV side, facilitating effective balloon contact and cryothermal energy delivery. Although catheter manipulation within the RV requires caution (e.g., gentle and stepwise catheter advancement under continuous fluoroscopic guidance) because of its smaller caliber, the anatomical size variability, and potential risk of mechanical injury, this case illustrates that the RV can serve as an effective anchoring site when conventional pulmonary vein anchoring is inadequate. This technique may serve as a useful bailout strategy for achieving durable LARB when conventional anchoring within the superior pulmonary veins is insufficient, such as in the presence of collapse toward the contralateral pulmonary vein. When LARB creation cannot be achieved using the cryoballoon alone, radiofrequency touch‐up ablation should be considered.

## Author Contributions

**Yuhei Kasai:** conceptualization, writing – original draft. **Takayuki Kitai:** supervision. **Junji Morita:** writing – review and editing. **Kei Murakami:** writing – review and editing. **Yumetsugu Munakata:** visualization. **Ryo Horita:** writing – review and editing. **Takuya Haraguchi:** writing – review and editing. **Jungo Kasai:** supervision, writing – review and editing. **Daisuke Hachinohe:** supervision.

## Funding

This study did not receive any funding.

## Ethics Statement

The examination was made in accordance with the approved principles. All the preparations and the equipment used are officially certified for the clinical use.

## Consent

Written informed consent was obtained from the patient for publication of this case report and any accompanying images.

## Conflicts of Interest

The authors declare no conflicts of interest.

## Data Availability

The data that support the findings of this study are available from the corresponding author upon reasonable request.

## References

[1] M. Kuniss , E. Akkaya , A. Berkowitsch , et al., “Left Atrial Roof Ablation in Patients With Persistent Atrial Fibrillation Using the Second‐Generation Cryoballoon: Benefit or Wasted Time?,” Clinical Research in Cardiology 109, no. 6 (2020): 714–724. 31667623 10.1007/s00392-019-01560-5

[2] S. Miyazaki , K. Hasegawa , M. Mukai , et al., “Cryoballoon Left Atrial Roof Ablation for Persistent Atrial Fibrillation‐Analysis With High‐Resolution Mapping System,” Pacing and Clinical Electrophysiology 45, no. 5 (2022): 589–597. 34427933 10.1111/pace.14345

[3] L. Lickfett , R. Kato , H. Tandri , et al., “Characterization of a New Pulmonary Vein Variant Using Magnetic Resonance Angiography: Incidence, Imaging, and Interventional Implications of the ‘Right Top Pulmonary Vein’,” Journal of Cardiovascular Electrophysiology 15, no. 5 (2004): 538–543. 15149422 10.1046/j.1540-8167.2004.03499.x

